# Grape Seed Procyanidin B2 Protects Porcine Ovarian Granulosa Cells against Oxidative Stress-Induced Apoptosis by Upregulating let-7a Expression

**DOI:** 10.1155/2019/1076512

**Published:** 2019-11-19

**Authors:** Jia-Qing Zhang, Xian-Wei Wang, Jun-Feng Chen, Qiao-Ling Ren, Jing Wang, Bin-Wen Gao, Zhi-Hai Shi, Zi-Jing Zhang, Xian-Xiao Bai, Bao-Song Xing

**Affiliations:** ^1^Henan Key Laboratory of Farm Animal Breeding and Nutritional Regulation, Institute of Animal Husbandry and Veterinary Science, Henan Academy of Agricultural Sciences, Zhengzhou 450002, China; ^2^Henan Provincial Animal Husbandry General Station, Zhengzhou 450008, China

## Abstract

Oxidative stress is a causal factor and key promoter of all kinds of reproductive disorders related to granulosa cell (GC) apoptosis that acts by dysregulating the expression of related genes. Various studies have suggested that grape seed procyanidin B2 (GSPB2) may protect GCs from oxidative injury, though the underlying mechanisms are not fully understood. Therefore, whether the beneficial effects of GSPB2 are associated with microRNAs, which have been suggested to play a critical role in GC apoptosis by regulating the expression of protein-coding genes, was investigated in this study. The results showed that GSPB2 treatment protected GCs from a H_2_O_2_-induced apoptosis, as detected by an MTT assay and TUNEL staining, and increased let-7a expression in GCs. Furthermore, let-7a overexpression markedly increased cell viability and inhibited H_2_O_2_-induced GC apoptosis. Furthermore, the overexpression of let-7a reduced the upregulation of Fas expression in H_2_O_2_-treated GCs at the mRNA and protein levels. Dual-luciferase reporter assay results indicated that let-7a directly targets the Fas 3′-UTR. Furthermore, the overexpression of let-7a enhanced the protective effects of GSPB2 against GC apoptosis induced by H_2_O_2_. These results indicate that GSPB2 inhibits H_2_O_2_-induced apoptosis of GCs, possibly through the upregulation of let-7a.

## 1. Introduction

Although numerous follicles are present in both ovaries 10 days after birth in sows, more than 99% of follicles undergo a degenerative process known as atresia during growth and development [1]. Recent studies have indicated that granulosa cell (GC) apoptosis plays a crucial role in the initiation of this process [2, 3]. Reactive oxygen species (ROS) are an inevitable byproduct of normal aerobic metabolism, nutrient deprivation, and environmental stimuli [4, 5]. In fact, ROS are necessary for fundamental cellular processes, such as cell proliferation, follicle development, and ovulation [6]. However, ROS generation exceeding the rate of ROS elimination leads to destructive effects on cellular components [7]. Previous studies have indicated that ROS accumulation in the ovary can give rise to GC apoptosis and antral follicle atresia in rats [8]. In mice, oxidative stress in vivo leads to GC apoptosis and follicular atresia [9]. In ewes, protein oxidation and GC apoptosis are increased, and GSH glutathione levels are decreased during follicular atresia [10]. In women, ROS scavenging efficiency in the follicular fluid leads to premature ovarian insufficiency and follicular atresia [11, 12]. In contrast, ROS inhibitors have been shown to alleviate GC injury in atretic follicles [13]. Therefore, the discovery and identification of an antioxidant by targeting oxidative stress-induced apoptosis may provide benefits for GC survival against oxidative injury.

MicroRNAs (miRNAs) are small, non-protein-coding RNAs that negatively regulate 30% of genes via degradation or posttranslational inhibition of their target mRNAs [14]. The abnormal expression of miRNAs has been corroborated in ovary-related diseases, such as polycystic ovary syndrome (PCOS) [15]. miRNAs play a central role in regulating the expression of key protein-coding genes related to GC apoptosis [16]. Moreover, numerous studies have indicated that miRNAs are involved in the regulation of apoptosis in response to oxidative stress [17–19]. let-7a is a member of the let-7 miRNA family and is specifically expressed in GCs [20]. Furthermore, recent studies have suggested that let-7a has an antiapoptotic effect, protecting endothelial cells and bronchial epithelial cells against apoptosis during stress [21, 22]. In addition, the level of let-7a in early atretic and progressively atretic ovary follicles is reduced compared with that in healthy follicles in sows [23]. These findings suggest that let-7a may play important roles in follicle development and atresia.

Oxidative stress-induced GC apoptosis is believed to be a major cause of follicular atresia [9]. Hence, the exploration of antiapoptosis measures is essential to alleviate GC injury in follicular development. In recent years, many natural plant extracts, such as resveratrol and curcumin, have been applied to alleviate oxidative stress and maintain the normal function of the ovary [24, 25]. Grape seed procyanidin B2 (GSPB2), an antioxidative and anti-inflammatory polyphenol in grape seed, has been reported to protect against ovarian oxidative damage [26, 27]. However, the exact mechanism by which GSPB2 protects GCs from oxidative stress has not been fully elucidated. Recently, some miRNAs have been reported to interfere with and modulate GC apoptosis signaling [16]. In this study, we used GSPB2 as an antioxidant to reduce oxidative stress levels and apoptosis in GCs exposed to H_2_O_2_ and focused on how the protective effects are modulated by miRNAs to identify a novel therapeutic target in porcine reproductive diseases.

## 2. Materials and Methods

### 2.1. Animals and Experimental Design

Twenty-four healthy gilts with similar body weight (40.00 ± 0.5 kg) (Duroc × Landrace × Large White) (Henan Agricultural Science and Technology Co., China) were randomly divided into four groups (*n* = 6): control group, grape seed procyanidin (GSP) group, diquat group, and GSP+diquat group. The gilts were housed in stalls, where they were fed twice daily and had free access to water. The environmental temperature was maintained at 24 ± 2°C. After 3 days of adaptation, the GSP and GSP+diquat groups were fed with GSP (200 mg/kg) for 2 consecutive days, followed by oxidative stress stimulation using a well-established in vivo model [28]. Briefly, on day 3, gilts received an additional intraperitoneal injection of diquat (10 mg/kg) or 0.9% saline. After injection of diquat, feed in the GSP and GSP+diquat groups was added 200 mg/kg GSP. After 7 days of experimental period, ovaries were collected surgically after each treatment. GCs were obtained from the left ovaries for subsequent miRNA and western blotting analysis. The right ovaries were fixed in 4% paraformaldehyde for the terminal deoxynucleotidyl transferase dUTP nick end labeling (TUNEL) assay. All animal experiments were performed in compliance with the Guidelines on the Humane Treatment of Laboratory Animals (Ministry of Science and Technology of the People's Republic of China, Policy No. 2006-398).

### 2.2. Measurement of the Oxidative Stress Index

Ovary tissue (150 mg) was homogenized in a tissue lyser for 1 min and then centrifuged for 10 min at 10,000 g at 4°C to remove the cellular debris; then, the supernatant was collected for oxidative stress index analysis. The activities of T-AOC, SOD, GSH-Px, and MDA concentrations in serum were measured using assay kits (Nanjing Jiancheng Bioengineering Research Institute, Nanjing, China) with Catalog nos. A015-2, A001-1, A005-1, and A003-2, respectively.

### 2.3. GC Culture and Treatment

Ovaries from mature 6-month-old healthy sows (Duroc × Landrace × Large White) were collected at a local slaughterhouse in Huiji District, Zhengzhou, China. The sows were housed in stalls, where they were fed twice daily and had free access to water. The environmental temperature was maintained at 24 ± 2°C. They were vaccinated against classical swine fever, foot-and-mouth disease, Aujeszky's disease, and porcine parvovirus. Before slaughter, electrodes were used to ensure that the pigs were in an unconscious state. After slaughter, the ovaries were removed and transported to the laboratory in a vacuum flask (33-35°C) containing sterile physiological saline (100 IU/mL penicillin and 100 *μ*g/mL streptomycin; Life Technologies) within 2-3 h of collection. In the laboratory, the ovaries were washed twice with sterile physiological saline. Hypodermic needles (25 gauge) were used to puncture 3-5 mm healthy follicles to collect GCs, which were washed twice with preheated phosphate-buffered saline (PBS, 37°C). The GCs were then seeded into T25 flasks and cultured at 37°C with 5% CO_2_ in a DMEM/F12 medium (Gibco) supplemented with 1% antibiotics (100 IU/mL penicillin and 100 *μ*g/mL streptomycin; Gibco) and 15% fetal bovine serum (FBS; Gibco). At 80-90% confluence, the GCs were washed with PBS once and subcultured with 0.25% trypsin-EDTA solution. Before the formal experiment, the GCs were exposed to a range of concentrations (from 0 *μ*mol/L to 500 *μ*mol/L) of H_2_O_2_ to induce oxidative damage. After examining the cell viability, caspase-3 activity, and apoptosis rates, we chose 200 *μ*M H_2_O_2_ for 6 h as the treatment conditions for the subsequent experiments. GSPB2 was purchased from Yuanye Inc. (>98% pure, batch no. 29106-49-8, Shanghai, China). The GCs were pretreated with GSPB2 (from 1 *μ*mol/L to 20 *μ*mol/L) supplemented with 5% FBS for 24 h, after which the medium was replaced with a medium containing 200 *μ*M H_2_O_2_ for 6 h.

### 2.4. RNA Preparation, Reverse Transcription, and Quantitative Real-Time (RT) PCR

Total RNA was extracted from GCs using TRIzol reagent (Invitrogen) following the manufacturer's protocol. The quantity and quality of the RNAs were measured using a NanoDrop 2000 (Thermo Scientific) and an Agilent 2100 Bioanalyzer (Agilent Technologies). Total RNA (1 *μ*g) was reverse transcribed in a final 20 *μ*L reaction volume using PrimeScript RT Master Mix (TaKaRa, Osaka, Japan). Then, qRT-PCRs were performed with a standard SYBR Green PCR kit (TaKaRa, Dalian, China) on a LightCycler 480 (Roche). Each reaction included 10 *μ*L of 2x SYBR Premix Ex Taq™, 1.0 *μ*L of each primer (10 *μ*mol/L), 2 *μ*L of cDNA, and 7 *μ*L of ddH_2_O. Triplicate samples were assessed for the selected target genes, and porcine GAPDH was used as an internal control. The relative expression levels of the selected target genes were determined by the 2-^*ΔΔ*Ct^ method. The primer sequences are listed in Supplemental [Supplementary-material supplementary-material-1]. The miRNA was extracted from the cell samples using a miRNA Extraction kit (HaiGene, Harbin, China) according to the manufacturer's protocol. Briefly, the reaction mix (20 *μ*L) consisting of 100 ng of miRNA, 5 *μ*L of 4x One Step miRNA RT Solution, and 2 *μ*L of 10x miRNA RT Primers was incubated at 37°C for 1 hour followed by 95°C for 5 min for enzyme inactivation. The quantification of miRNA was performed by an HG miRNA SYBR Green PCR kit (HaiGene, Harbin, China). Real-time PCR was performed in a 20 *μ*L volume with 1 *μ*L of cDNA, 1 *μ*L of each primer (10 *μ*mol/L), 14 *μ*L of ddH_2_O, and 4 *μ*L of Golden HS SYBR Green qPCR Mix under the following conditions: 95°C for 15 min, 35 cycles of 95°C for 5 s, and 60°C for 30 s in a LightCycler 480 (Roche). let-7a levels were normalized to U6B RNA levels using the 2−^*ΔΔ*Ct^ method. The miRNA primers for let-7a were based on those in a previously published study [29].

### 2.5. Transfections

A let-7a mimic (let-7a mi), let-7a inhibitor (let-7a in), scrambled oligonucleotides negative control (NC), Fas siRNAs, and control siRNA (NC-siRNA) were purchased from Shanghai GenePharma (Supplementary [Supplementary-material supplementary-material-1]). GCs were cultured in culture dishes to 70%-80% confluence. The let-7a mimics, inhibitor, NC, or Fas siRNA or control siRNA were transfected into GCs using Lipofectamine 2000 reagent (Invitrogen, Carlsbad, CA, USA), according to the manufacturer's instructions. The transfected GCs were used for subsequent experiments or analysis 48 h after transfection.

### 2.6. Apoptosis Analysis

GCs transfected with the let-7a mimic, NC, Fas siRNA, or siRNA NC were harvested 48 h after transfection. The apoptotic effect was measured using a TUNEL Detection kit (Roche Applied Science). The detailed procedure was performed according to the manufacturer's instructions. The Olympus IX-73 fluorescence microscope (Olympus, Tokyo, Japan) was used to obtain fluorescence images. The total apoptotic cell number and the total cell number were counted in five fields of vision; the apoptosis rate was then calculated.

### 2.7. Dual-Luciferase Reporter Assay

Bioinformatics prediction is a powerful tool for the study of the functions of miRNAs. TargetScan (http://www.targetscan.org/) and miRanda (http://www.microrna.org/microrna/home.do) were used to predict let-7a target genes. Bioinformatics analyses identified a putative let-7a binding site at positions (nt 503-nt 509) of the Fas 3′-UTR, and the mfe was -19.6 kcal/mol. To determine whether Fas is a direct target of let-7a, luciferase activity assays were performed using luciferase reporters. The predicted let-7a miRNA-binding regions in the 3′-UTR of Fas were subcloned into the GP-miRGLO Luciferase miRNA expression vector (GenePharma, Shanghai, China). Mutants of the binding sites were used as NC. Two copies of the anti-let-7a oligonucleotide sequence were cloned into the pmirGLO vector, resulting in an ssc-let-7a inhibitor sponge (PC). The cloned product was confirmed by sequencing. HEK293T cells were plated in 24-well plates at 10^4^ cells per well and cotransfected with GP-miRGLO plasmids containing either the wild-type (WT) or mutated (MT) Fas 3′-UTR or the let-7a inhibitor plasmid, let-7a mimics, or NC. Firefly and Renilla luciferase activities were measured 48 h after transfection using the Dual-Luciferase Reporter Assay system (Promega, Madison, WI, USA).

### 2.8. GC Viability Assay

The viability of GCs was measured by MTT assay kits (Nanjing Jiancheng Bioengineering Institute, Nanjing, China) according to the manufacturer's instructions. Briefly, the GCs were treated as described above (transfection, H_2_O_2_ treatment, etc.) for the indicated times. Next, the GCs were treated with 20 *μ*L of 3-(4, 5-dimethylthiazol-2-yl)-2, 5-diphenyltetrazolium bromide (MTT; 0.5 mg/mL) for 4 h at 37°C. The medium was discarded and replaced with 150 *μ*L of dimethyl sulfoxide (DMSO). After 10 min of rotation, the absorbance was measured at 570 nm using a microplate reader (Bio-Rad, Hercules, CA, USA).

### 2.9. Caspase-3 Activity Assay

As a marker of apoptosis, caspase-3 activity in GCs was determined by colorimetric assay kits (Beyotime Institute of Biotechnology, China) according to the manufacturer's instructions. In brief, GCs were lysed, and the assay was performed by incubating 10 *μ*g of total protein in 100 *μ*L of reaction buffer containing caspase-3 substrate and incubated for 2 h at 37°C. Caspase-3 activity was detected by a microplate reader (Bio-Rad, Hercules, CA, USA) at 405 nm.

### 2.10. Western Blot Analysis

Primary antibodies against Fas (Cell Signaling Technology, Danvers, MA, USA) and *β*-actin (Cell Signaling Technology, Danvers, MA, USA) were used in the study. Western blotting was performed as described previously [26]. Protein bands were visualized by exposure to an enhanced chemiluminescence detection system (LAS-4000 imager, Fujifilm, Tokyo, Japan) following the manufacturer's instructions. The density of the protein bands was measured using the ImageJ software (US National Institutes of Health). The gray value of each target protein was normalized to that of *β*-actin before comparison.

### 2.11. Statistical Analyses

Statistical analyses were performed using SPSS v18.0 software (SPSS, Chicago, IL, USA). The results are presented as mean ± standard error (S.E.) from at least three independent experiments. *P* values less than 0.05 and 0.01 were considered as significant and extremely significant differences, respectively.

## 3. Results

### 3.1. Effects of GSP on the Antioxidant Capacity in the Diquat-Induced Ovarian Tissue

**Figure 1:**
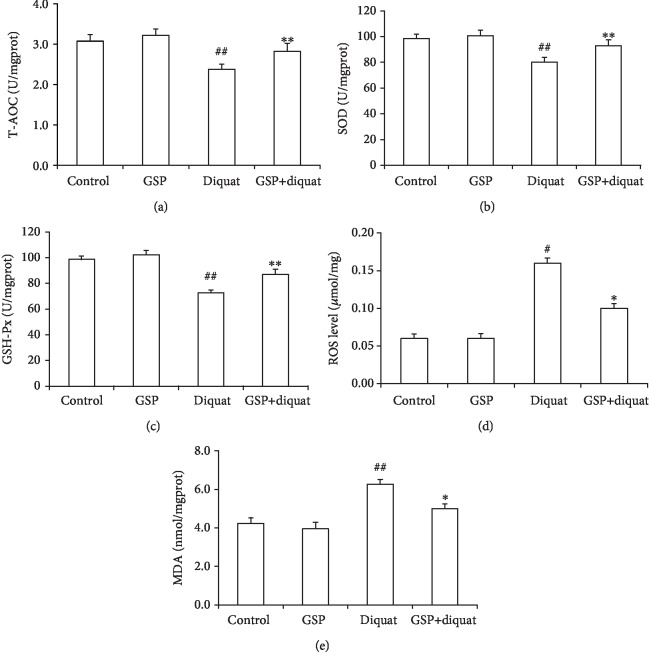
Effects of GSP on antioxidant status in diquat-induced ovarian oxidative injury. (a) Effects of GSP on the total antioxidant capacity (T-AOC) activity. (b) Effects of GSP on the superoxide dismutase (SOD) activity. (c) Effects of GSP on the glutathione peroxidase (GSH-Px) activity. (d) Effects of GSP on the malondialdehyde (MDA) content. (e) Effects of GSP on the reactive oxygen species (ROS) levels. Data was indicated as the mean ± S.E. (*n* = 3). ^#^*P* < 0.05, ^##^*P* < 0.01 diquat alone group vs. the control; ^∗^*P* < 0.05, ^∗∗^*P* < 0.01 vs. the diquat alone group.

Diquat has been widely used to induce oxidative stress in vivo [28, 30]. In order to determine the effect of diquat alone or combined with GSP on the antioxidant capacity of ovarian tissues, the activities of antioxidant enzymes (T-AOC, SOD, and GSH-Px), ROS levels, and MDA contents in the ovarian tissues from four groups were detected. The results indicated that the activities of T-AOC, SOD, and GSH-Px were markedly decreased and the ROS levels and MDA contents were significantly increased in the ovarian tissue treated with diquat compared with those in the control group (Figures 1(a)–1(e)). However, these trends were attenuated by simultaneous GSP supplement. These results showed that the antioxidant capacity of ovarian tissues decreased after diquat treatment and GSP could inhibit this reduction.

### 3.2. Effects of GSP on the Diquat-Induced GC Apoptosis in Antral Follicles

**Figure 2:**
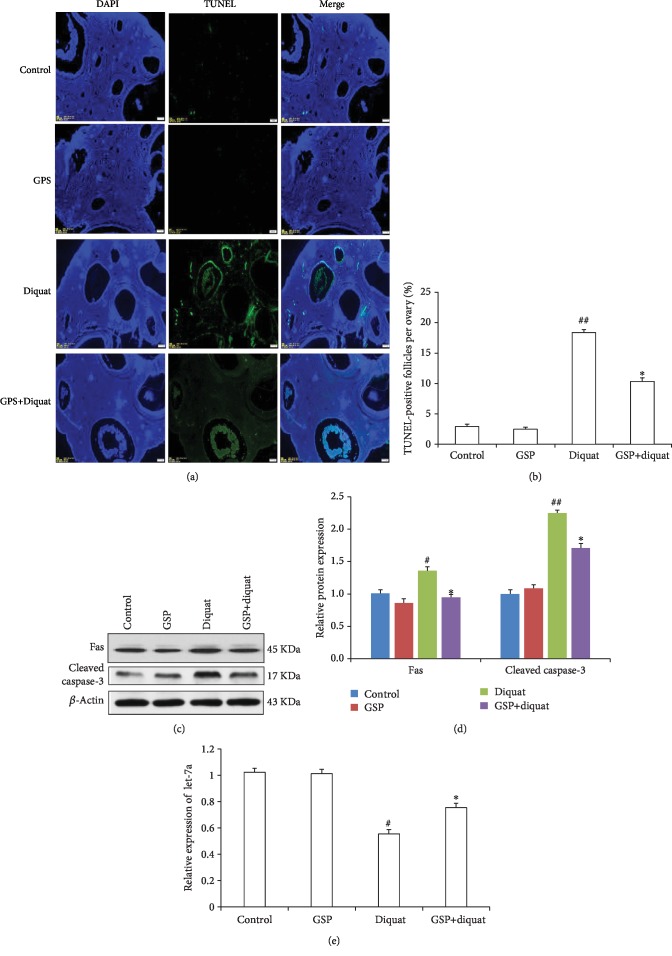
Effects of GSP on the diquat-induced GC apoptosis in antral follicles. (a) TUNEL assay of GC apoptosis in the porcine ovary sections. (b) Quantification of TUNEL-positive antral follicles. (c) Relative expression of proteins related to GC apoptosis. (d) Quantification of relative Fas and cleaved caspase-3 protein levels. (e) The relative expression level of let-7a was analyzed by RT-PCR. Data was indicated as the mean ± S.E. (*n* = 3). ^#^*P* < 0.05, ^##^*P* < 0.01 diquat alone group vs. the control; ^∗^*P* < 0.05 vs. the diquat alone group.

The ovaries from control and treated animals were embedded in paraffin for the TUNEL assay. The results showed that the number of TUNEL-positive follicles was significantly increased in the ovary treated with diquat compared with that in the control group (Figures 2(a) and 2(b)). Furthermore, the percentage of TUNEL-positive follicles was significantly reduced in the GSP supplementation group. Moreover, diquat treatment significantly upregulated the expression levels of Fas and cleaved caspase-3, but these changes were attenuated by GSP supplementation (Figures 2(c) and 2(d)). let-7a has been reported to be involved in the regulation of GC apoptosis. To determine whether GSP modulates the expression of let-7a in GCs, the expression level of let-7a from four groups was determined by RT-PCR. As shown in Figure 2(e), the GCs in the diquat-treated group showed a decrease in the expression of let-7a. However, GSP and diquat cotreatment significantly upregulated the expression of let-7a. These results indicated that GSP supplement may inhibit the diquat-induced increase in GC apoptosis and decrease in let-7a expression.

### 3.3. GSPB2 Protects GCs from H_2_O_2_-Induced Apoptosis

**Figure 3:**
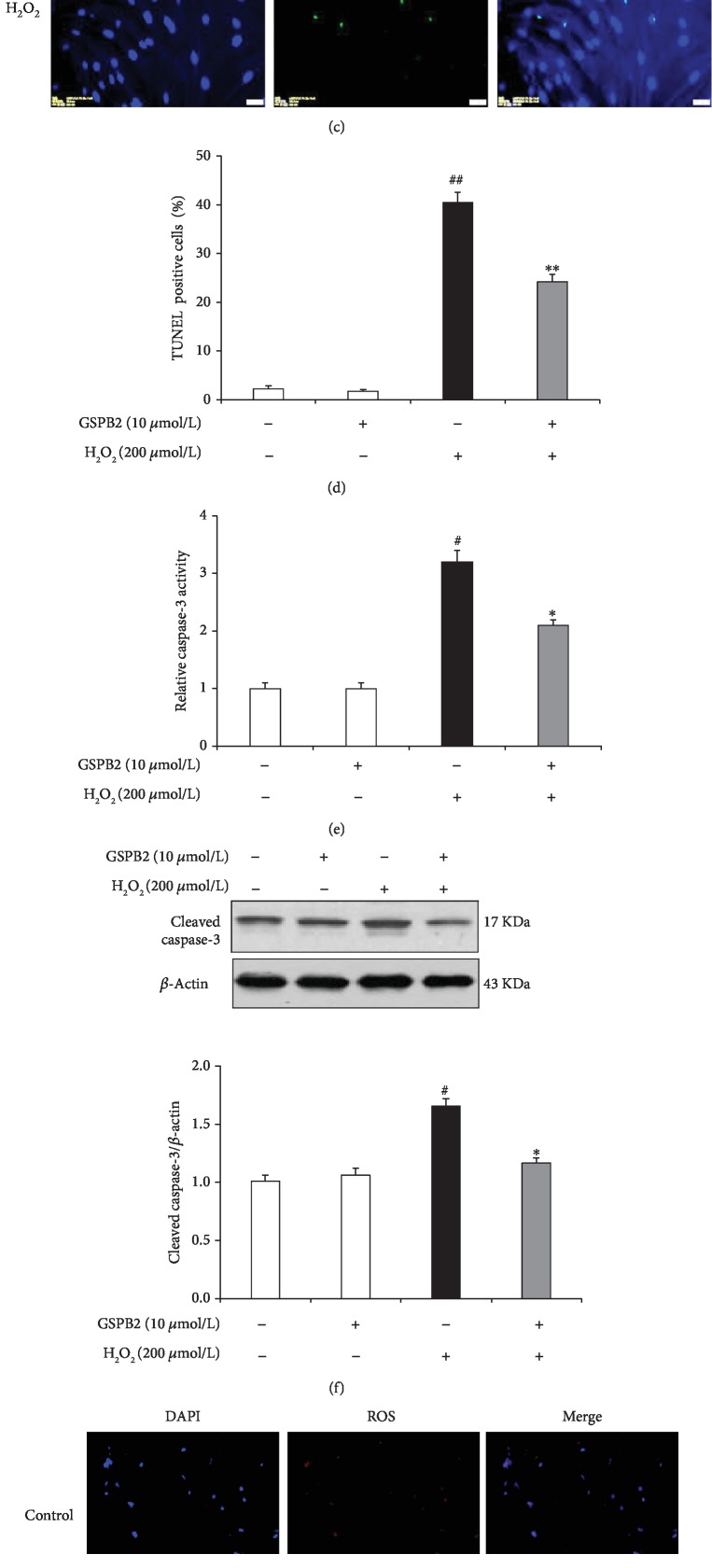
The protective effects of GSPB2on H_2_O_2_-induced cytotoxicity in GCs. (a) GC survival was tested following treatment with a range of H_2_O_2_ concentrations (50, 100, 200, 300, and 500 *μ*mol/L). (b) Effects of GSPB2 on the viability of GCs treated with H_2_O_2_. GCs were exposed to various concentrations of GSPB2 (1, 5, 10, and 20 *μ*mol/L) for 24 h. Then, the GCs were treated with H_2_O_2_ (200 *μ*mol/L) for 6 h. (c, d) Treated GCs were subjected to TUNEL and DAPI staining. (c) Representative TUNEL staining. (d) Quantification of the apoptosis rates. (e) The activity of caspase-3 in GCs was measured by ELISA. (f) Western blot analysis of cleaved caspase-3. (g) Intracellular ROS levels were assessed by dihydroethidium bromide fluorescence. (h) Quantification of intracellular ROS levels. Each value is expressed as the mean ± S.E. (*n* = 3). ^#^*P* < 0.05, ^##^*P* < 0.01 H_2_O_2_ alone group vs. the control; ^∗^*P* < 0.05, ^∗∗^*P* < 0.01 vs. the H_2_O_2_ alone group.

GC viability was assayed following treatment with a range of concentrations (from 50 *μ*mol/L to 500 *μ*mol/L) of H_2_O_2_, and H_2_O_2_ was found to significantly reduce GC viability in a dose-dependent manner (Figure 3(a)). Based on the cell viability, treatment with 200 *μ*M H_2_O_2_ for 6 h was chosen as the optimum concentration for use in subsequent experiments. To evaluate whether GSPB2 protects GCs against oxidative damage, the MTT method was used to determine cell viability (Figure 3(b)). The treatment of GCs with 200 *μ*M H_2_O_2_ for 6 h significantly reduced GC viability, whereas GSPB2 (10 *μ*mol/L) inhibited H_2_O_2_-induced damage, restoring GC survival following H_2_O_2_ treatment. Moreover, GCs pretreated with GSPB2 (10 *μ*mol/L) significantly decreased the number of TUNEL-positive cells (Figures 3(c) and 3(d)).

Caspase-3 is a principal effector caspase in apoptotic cascades. Therefore, we analyzed the activity of caspase-3 in GCs and found that H_2_O_2_-induced stress significantly increased caspase-3 activity in treated cells compared with control cells (*P* < 0.05). The pretreatment of GCs with GSPB2 significantly suppressed H_2_O_2_-induced caspase-3 activity (Figure 3(e)). Furthermore, the protein expression of cleaved caspase-3 was markedly higher in H_2_O_2_-treated GCs than in H_2_O_2_-free group (Figure 3(f)). Moreover, H_2_O_2_-treated GCs displayed a high level of ROS accumulation, whereas the ROS level of the GSPB2 pretreated group appeared to decline dramatically (Figures 3(g) and 3(h)). These results suggest that 10 *μ*mol/L GSPB2 decreases oxidative stress-induced apoptosis and caspase-3 activity in cultured GCs.

### 3.4. GSPB2 Inhibits the H_2_O_2_-Induced Downregulation of let-7a Expression in GCs

**Figure 4:**
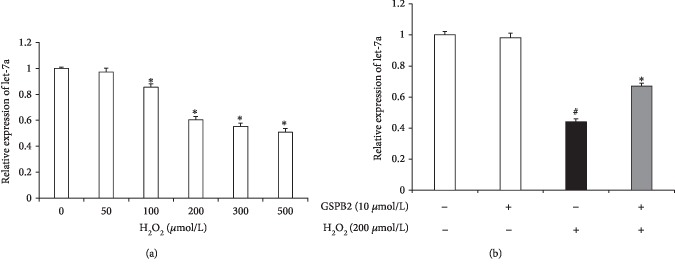
Effects of GSPB2 on the H_2_O_2_-induced downregulation of let-7a expression in GCs. (a) The expression levels of let-7a in cultured GCs were measured after 6 h of exposure to 0, 50, 100, 200, 300, and 500 *μ*mol/L H_2_O_2_. (b) GCs were pretreated with GSPB2 (10 *μ*mol/L) for 24 h followed by H_2_O_2_ (200 *μ*mol/L) for 6 h. The mRNA expression level of let-7a was analyzed by RT-PCR. Each value is expressed as the mean ± S.E. (*n* = 3). ^#^*P* < 0.05 H_2_O_2_ alone group vs. the control; ^∗^*P* < 0.05 vs. the H_2_O_2_ alone group.

let-7a has been suggested to modulate and interfere with apoptosis signaling. To determine whether H_2_O_2_-induced apoptosis affects the expression of endogenous let-7a in GCs, the cells were exposed to a range of concentrations of H_2_O_2_ for 6 h, and then, the expression of let-7a was determined by qRT-PCR. As shown in Figure 4(a), the let-7a levels were reduced in a dose-dependent manner. To determine whether GSPB2 affects the H_2_O_2_-induced expression of endogenous let-7a in GCs, the cells were pretreated with 10 *μ*M GSPB2 for 24 h followed by 200 *μ*M H_2_O_2_ for 6 h, and the expression of let-7a was determined by qRT-PCR. As shown in Figure 4(b), GSPB2 pretreatment significantly inhibited the H_2_O_2_-induced downregulation of let-7a expression in GCs (*P* < 0.05). These results show that GSPB2 inhibits H_2_O_2_-induced GC apoptosis by regulating let-7a expression.

### 3.5. Role of let-7a in GSPB2-Mediated Protection against H_2_O_2_-Induced Apoptosis and Caspase-3 Activity in GCs

**Figure 5:**
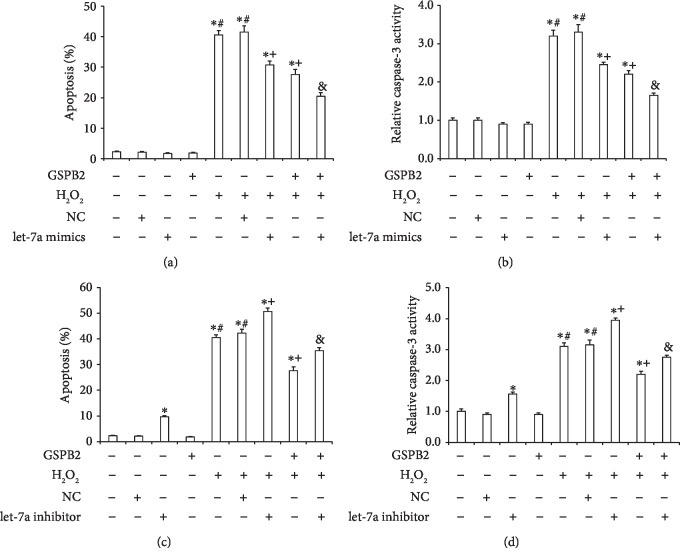
Effect of let-7a on GC apoptosis and caspase-3 activity. GCs were transfected with let-7a mimics and/or pretreated with GSPB2 and subsequently treated with H_2_O_2_ (200 *μ*mol/L) for 6 h. (a) The number of apoptotic cells was identified by the TUNEL method. (b) The levels of caspase-3 activity were measured by ELISA. GCs were transfected with let-7a inhibitors and/or pretreated with GSPB2 and subsequently treated with H_2_O_2_ (200 *μ*mol/L) for 6 h. (c) The apoptosis number in different groups was determined by the TUNEL method. (d) The caspase-3 activity in different groups was measured by ELISA. Each value is expressed as the mean ± S.E. (*n* = 3). ^∗^*P* < 0.05 vs. the control; ^#^*P* < 0.05 vs. the antioxidant pretreatment; ^+^*P* < 0.05 vs. H_2_O_2_; ^&^*P* < 0.05 vs. the GSPB2 pretreatment and H_2_O_2_.

To assess the role of let-7a in response to H_2_O_2_ and GSPB2 treatments, apoptosis and caspase-3 activity were examined in GCs in the presence or absence of let-7a mimics or inhibitors. The results of the apoptosis analysis indicated that the transfection of the let-7a mimics resulted in the negative regulation of GC apoptosis induced by H_2_O_2_ (*P* < 0.05) (Figure 5(a)). Furthermore, the caspase-3 activity results suggested that transfection of the let-7a mimics caused the negative regulation of H_2_O_2_-induced caspase-3 activity in GCs (*P* < 0.05) (Figure 5(b)). In contrast, transfection of the GCs with the let-7a inhibitor resulted in the positive regulation of the H_2_O_2_-induced apoptosis (*P* < 0.05) (Figure 5(c)). Moreover, transfection of the let-7a inhibitors positively regulated the caspase-3 activity of GCs induced by H_2_O_2_ (*P* < 0.05; Figure 5(d)). These data suggest that let-7 may act as a negative modulator of H_2_O_2_-induced apoptosis in GCs and that GSPB2 inhibits H_2_O_2_-induced apoptosis by upregulating the let-7a expression in GCs.

### 3.6. let-7a Overexpression Suppresses the H_2_O_2_-Induced Upregulation of Fas and Caspase-3 Expression in GCs

**Figure 6:**
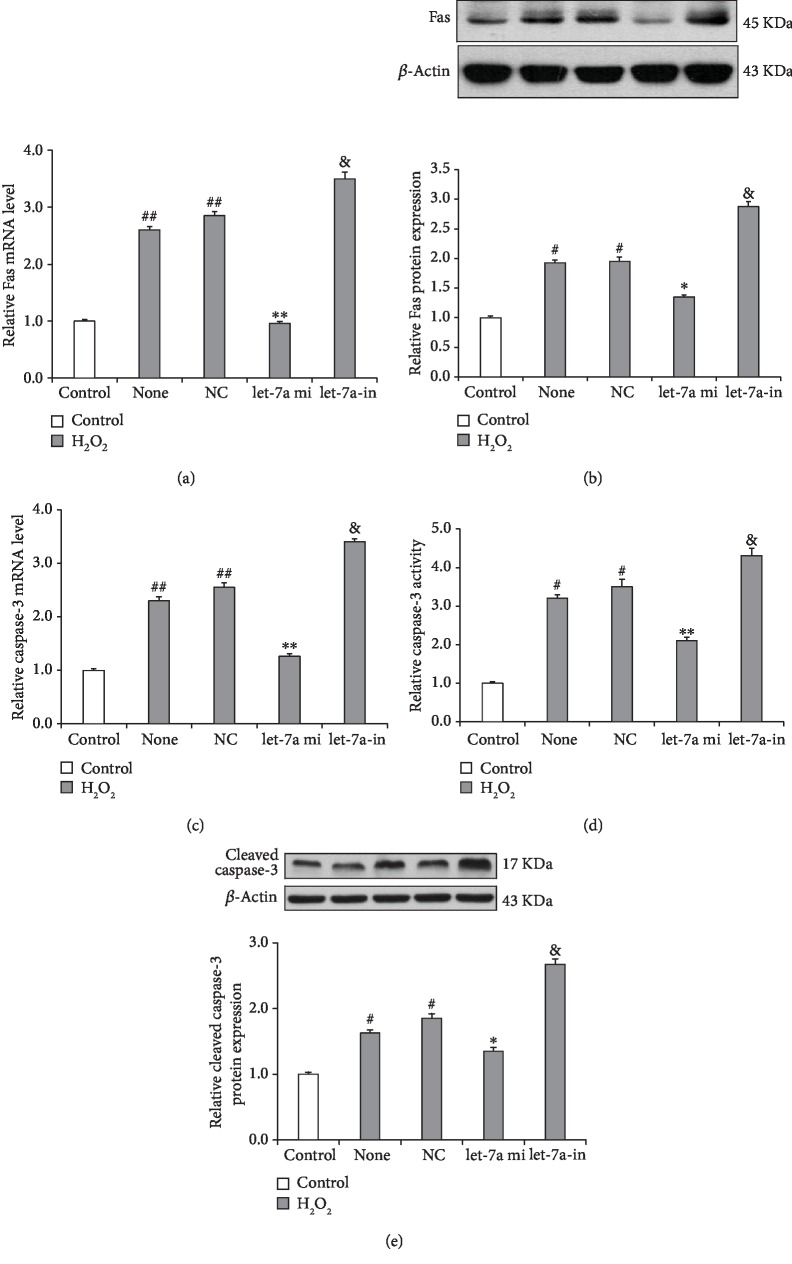
let-7a overexpression inhibits the expression of Fas and caspase-3. (a, b) The mRNA and protein expression levels of Fas were significantly upregulated by H_2_O_2_ but downregulated by let-7a overexpression. (c. d) The mRNA expression and caspase-3 activity were also markedly elevated by H_2_O_2_ but reduced by let-7a overexpression. (e) Western blot analysis of cleaved caspase-3. Each value is expressed as the mean ± S.E. (*n* = 3). ^#^*P* < 0.05, ^##^*P* < 0.01 vs. the H_2_O_2_-free group (control); ^∗^*P* < 0.05, ^∗∗^*P* < 0.05 vs. NC.

As Fas has been shown to activate caspase-3 activity [31], we further measured Fas and caspase-3 expression and caspase-3 activity in GCs overexpressing let-7a. As shown in Figures 6(a) and 6(b), Fas mRNA and protein expression was significantly upregulated in the H_2_O_2_-treated GCs compared to the control GCs, and this upregulation was reversed by let-7a overexpression. In contrast, transfection of the let-7a inhibitors increased Fas mRNA and protein expression in the H_2_O_2_-treated GCs. Furthermore, caspase-3 mRNA expression and activity were increased in the H_2_O_2_-treated GCs compared to the control GCs; this increased expression could be significantly reduced by let-7a overexpression. Furthermore, the caspase-3 mRNA expression and activity induced by H_2_O_2_ in the let-7a knockdown group were significantly elevated compared to those in the control group (Figures 6(c) and 6(d)). In addition, the cleaved caspase-3 protein expression induced by H_2_O_2_ in the let-7a overexpression and knockdown groups also showed the similar trend with the caspase-3 activity (Figure 6(e)). These results imply that Fas may be a downstream target of let-7a.

### 3.7. let-7a Directly Targets at the 3′-UTR of Fas

**Figure 7:**
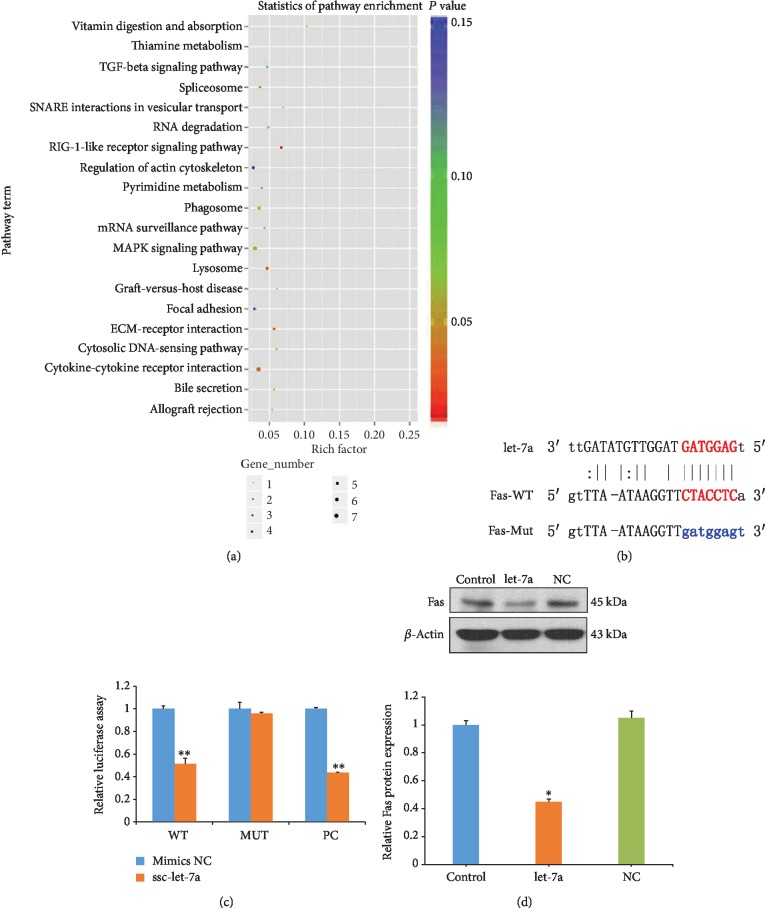
let-7a binds to the 3′-UTR of Fas and regulates gene expression. (a) Pathway enrichment analysis of let-7a targets. (b) The putative let-7a-binding sites in the porcine Fas 3′-UTR (red letters). The blue letters show the region that was mutated in the experiment shown in (b). (c) A dual-luciferase reporter assay was performed by cotransfection of HEK293T with luciferase reporters containing a WT or mutant 3′-UTR of porcine Fas with a let-7a mimic. (d) The Fas protein expression level was normalized to that of *β*-actin. Each value is expressed as the mean ± S.E. (*n* = 3). ^∗^*P* < 0.05, ^∗∗^*P* < 0.01 vs. the control group.

The predicted target genes of let-7a are listed in Supplementary [Supplementary-material supplementary-material-1]. The pathway analysis indicated that let-7a targets are enriched in the thiamine metabolism, TGF-*β*, and MAPK pathways, as well as in some other pathways in the KEGG database (Figure 7(a)). The related gene ontology terms were also analyzed based on let-7a targets. Fas was identified as a putative let-7a target; Fas is widely expressed in porcine ovary GCs, and its mRNA and protein levels in GCs are upregulated during follicular atresia [32, 33]. Therefore, Fas was chosen as a candidate target gene for further study. Computer-based sequence analysis software identified an eight-mer putative let-7a binding site in the 3′UTR of the Fas gene (Figure 7(b)). To determine whether Fas is a direct target of let-7a, we cotransfected HEK293T cells with Fas 3′UTR-WT or Fas 3′UTR-MT with the let-7a mimic or NC and measured the relative luciferase activity. The results indicated that the let-7a mimic, but not the NC, significantly inhibited the luciferase activity of the Fas 3′UTR-WT but not the Fas 3′UTR-MT, demonstrating that let-7a could target the 3′-UTR of Fas (Figure 7(c)). Furthermore, western blot analyses showed that let-7a overexpression inhibited the expression of Fas (Figure 7(d)). Taken together, these results indicate that Fas is targeted by let-7a in the inhibition for GC apoptosis.

### 3.8. Fas Is Involved in the GSPB2-Mediated Protection against H_2_O_2_-Induced Apoptosis

**Figure 8:**
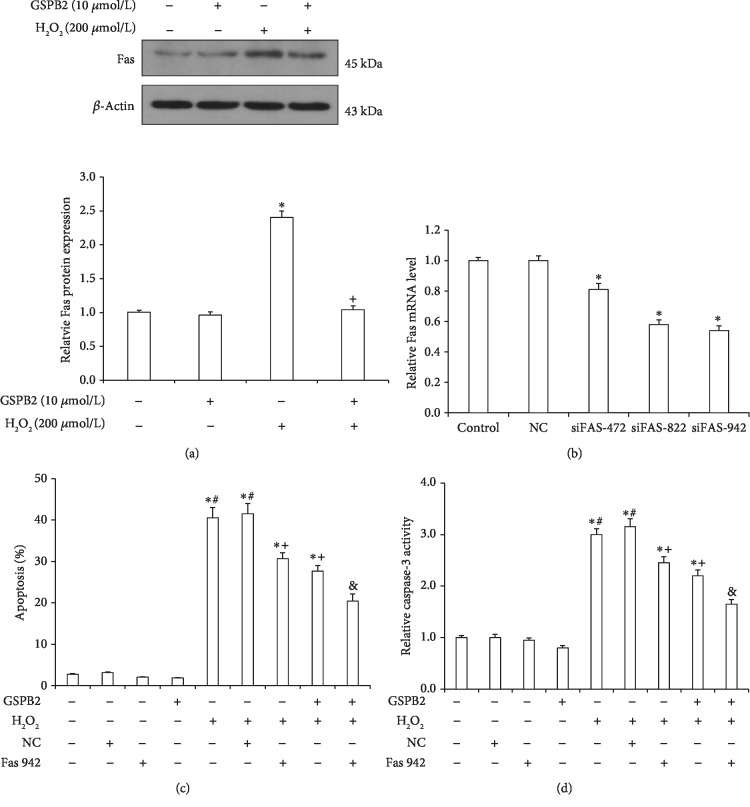
Effects of Fas knockdown on the GSPB2-mediated protection of GCs against H_2_O_2_-induced apoptosis. GCs were pretreated with GSPB2 and then treated with H_2_O_2_ (200 *μ*mol/L) for 6 h. (a) The protein expression level of Fas was analyzed by western blotting. (b) Fas knockdown was verified by qRT-PCR. Fas siRNA-transfected GCs were pretreated with GSPB2 and then treated with H_2_O_2_ (200 *μ*mol/L) for 6 h. (c) The levels of apoptosis were measured by the TUNEL method. (d) The levels of caspase-3 activity were measured by ELISA. Each value is expressed as the mean ± S.E. of three independent experiments. ^∗^*P* < 0.05 vs. the control; ^#^*P* < 0.05 vs. GSPB2 pretreatment; ^+^*P* < 0.05 vs. H_2_O_2_; ^&^*P* < 0.05 vs. GSPB2 pretreatment and H_2_O_2_.

Next, we explored whether the counteraction of the GSPB2-mediated protection against H_2_O_2_-induced apoptosis by let-7a overexpression is mediated by Fas. The protein expression of Fas in GCs treated with H_2_O_2_ for 6 h after pretreatment with GSPB2 for 24 h was analyzed by western blotting. In addition, the apoptosis and caspase-3 activity of Fas-knockdown cells treated with H_2_O_2_ for 6 h after pretreatment with GSPB2 for 24 h were assessed. The protein expression of Fas in GCs after H_2_O_2_ treatment for 6 h was significantly increased compared with that in the control GCs, and GSPB2 pretreatment for 24 h decreased the expression of Fas in GCs exposed to H_2_O_2_ (Figure 8(a)). Real-time PCR indicated that the expression levels of the Fas mRNA were significantly lower in GCs transfected with siRNA than those transfected with the NC siRNA (*P* < 0.05; Figure 8(b)). Furthermore, the knockdown of Fas clearly promoted the protective effect of GSPB2 against H_2_O_2_-induced apoptosis and caspase-3 activity in GCs (*P* < 0.05; Figures 8(c) and 8(d)). These results show that Fas is involved in the protective effect of GSPB2-mediated protection against H_2_O_2_-induced apoptosis in GCs.

## 4. Discussion

In the mammalian ovary, the majority of follicles (more than 99%) undergo a degenerative process known as atresia [32]. Numerous studies have shown that GC apoptosis plays a key role in the regulation of ovarian follicular atresia [26, 34, 35]. Antral follicles are inevitably exposed to oxidative stress produced by ROS during development. We previously confirmed that ROS-induced GC apoptosis is a key cause of follicular atresia [26, 36]. Furthermore, a growing body of evidence suggests that excessive ROS induce the initiation of GC apoptosis, resulting in antral follicular atresia [9, 26]. Moreover, ROS-induced GC apoptosis plays a central role in certain anovulatory disorders, such as premature ovarian failure and polycystic ovary syndrome [6]. Therefore, to inhibit ROS-induced GC apoptosis and the associated female reproductive diseases, it is crucial to clarify the molecular mechanisms that regulate GC apoptosis and identify potential therapeutic targets. Oxidative stress can trigger apoptosis through three main signaling pathways: (1) the extrinsic or death receptor pathway, (2) the intrinsic or mitochondrial pathway, and (3) the endoplasmic reticulum pathway [37]. In the death receptor pathway, transmembrane death receptors such as Fas, TRAIL-R1/2, and TNF-R1 can be activated by ROS. This activation results in the activation of caspases-8/10, which can directly activate caspases-3/6/7 and trigger apoptosis [2]. In the mitochondrial pathway, ROS generated exogenously or endogenously can elevate the ratio of proapoptotic proteins (Bad, Bax)/antiapoptotic proteins (Bcl-2) expression, resulting in mitochondrial membrane potential reduction, cytochrome c release, and caspase-induced apoptosis [9]. In the endoplasmic reticulum, ROS stimulate the activation of PERK, ATF-6*α*, and IRE1*α*. The activation of PERK and ATF-6*α* induces the expression of CHOP, which activates apoptosis by upregulating the expression of proapoptotic genes such as Bax and Bim and/or by inhibiting the expression of the Bcl-2 gene [37]. Many studies have indicated that miRNAs regulate the expression of GC apoptotic genes [38]. Fas activation-induced GC apoptosis is a critical mediator of follicular atresia [39]. The results of the present study indicate that let-7a levels are significantly decreased during H_2_O_2_ exposure-induced GC apoptosis. let-7a overexpression reduces caspase-3 activity and the percentage of apoptotic cells after H_2_O_2_ exposure. Furthermore, let-7a overexpression decreases Fas mRNA and protein levels in GCs by directly targeting the Fas 3′UTR. Fas expression level is upregulated in GCs after H_2_O_2_ treatment, and Fas knockdown dramatically decreases caspase-3 activity and the apoptotic cell percentage. These results show that decreases in let-7a expression and increases in Fas expression resulting from H_2_O_2_ exposure may increase GC oxidative damage, caspase-3 activity, and the apoptotic cell percentage.

Our findings suggest that let-7a expression is dramatically downregulated in GCs after H_2_O_2_ treatment. This result is consistent with those of previous studies [22, 40]. Furthermore, let-7a miRNA expression is most markedly decreased in early and progressively atretic porcine follicles [23], indicating that it might be associated with the pathogenesis of H_2_O_2_-induced GC oxidative damage and apoptosis. Therefore, we examined the role of let-7a expression in H_2_O_2_-induced GC apoptosis. Using bioinformatics, dual-luciferase assays, and protein expression level analyses, we found that Fas is a direct target of let-7a. The Fas/FasL system is the most characterized cell death ligand-receptor system. Fas and FasL are expressed in porcine, bovine, and murine ovaries and play a key role in the regulation of GC apoptosis [32]. In murine ovaries, Fas has been observed in the GCs of atretic antral follicles [39]. In porcine ovarian follicles, the expression levels of Fas mRNA and protein are increased in the GCs during follicular atresia [32]. In bovine ovaries, Fas is scattered in the GC layer of atretic follicles. The results herein indicate that Fas expression is markedly increased in H_2_O_2_-induced GC apoptosis. Furthermore, let-7a overexpression dramatically reverses the expression of Fas mRNA and protein. Caspase-3 plays a pivotal role in the execution phase of stress-induced cell apoptosis [41]. Furthermore, caspase-3 is considered a dominant executor in Fas activation-induced apoptosis [42, 43]. Thus, the inhibition of caspase-3 activity may inhibit GC apoptosis. In this study, we found that caspase-3 activity was markedly decreased by let-7a overexpression. Moreover, Fas knockdown dramatically downregulated H_2_O_2_-induced caspase-3 activity in GCs. Furthermore, Fas knockdown by siRNA protected GCs from oxidative injury and apoptosis in GCs exposed to H_2_O_2_. These data show that Fas is a downstream target of let-7a and that it mediates the effect of let-7a on GC oxidative injury and apoptosis after H_2_O_2_ treatment.

Accumulating data demonstrate that the antioxidant supplementation is an efficient measure to dampen ovarian oxidative damage [26, 27, 44]. GSPB2 is one of the main components of proanthocyanidin extracts from grape seed and has been shown to be a potent antioxidant and effective scavenger of free radicals [45]. Recent studies have shown that grape seed proanthocyanidin extract (GSPE) alleviates oxidative damage in mouse testis and liver by activating Nrf2 signaling [46, 47]. Another study demonstrated that GSPE can reduce high-fat-diet- (HFD-) induced dyslipidemia in mice by attenuating the HFD-induced upregulation of miR-96 expression [48]. We previously found that GSPB2 alleviates oxidative stress-induced GC apoptosis by evoking an autophagic response [26]. In addition, GSPB2 treatment during in vitro culture decreases apoptosis and ROS generation in various cell types [45, 49]. In the present study, we investigated the potential of GSPB2 to alleviate the oxidative stress-induced porcine GC apoptosis.

Our results show that pretreatment with GSPB2 reduces GC apoptosis and caspase-3 activity induced by oxidative stress. Furthermore, we found marked decreases in the apoptotic cell percentage and caspase-3 activity when GCs were pretreated with GSPB2 24 h after H_2_O_2_ treatment for 6 h. These findings are consistent with those of a study by Xiang et al., who found that anthocyanins inhibit ROS-induced apoptosis and oxidative stress in porcine GCs [50]. Our results also show that a decrease in the apoptotic cell percentage and caspase-3 activity by GSPB2 might represent a novel treatment strategy to increase conception rates and reproductive efficiency. Additionally, several recent reports have shown that miRNA participates in ROS generation and apoptosis signaling during follicular atresia [19, 51]. Here, we aimed to illuminate the biological role of let-7a in the regulation of porcine GC apoptosis by H_2_O_2_ and GSPB2. The data demonstrated a significant decrease in the let-7a expression in GCs treated with H_2_O_2_, but an increase in let-7a expression in GCs treated with GSPB2 before H_2_O_2_ treatment. Moreover, the knockdown of the let-7a target gene Fas or let-7a overexpression phenocopied the protective effects of GSPB2 on H_2_O_2_-induced apoptosis and reduced the apoptosis rate and caspase-3 activity. These data support the hypothesis that the protective effects of GSPB2 against H_2_O_2_-induced porcine GC apoptosis are partially mediated by an increase in let-7a expression.

In conclusion, our findings show that let-7a reduces porcine GC apoptosis, reduces the caspase-3 activity, and improves the porcine GC viability. The protective effect of let-7a in GCs is achieved at least partially through the downregulation of the activity of the Fas/caspase-3 apoptotic signaling pathway. This finding implies that let-7a overexpression during ROS-induced GC apoptosis might be a useful approach for the treatment of ovarian dysfunction and infertility.

## Data Availability

The data used to support the findings of this study are available from the corresponding authors upon request.

## Supplementary Materials

Supplementary 1 Table S1: the forward and reverse primers used for qRT-PCR.

Supplementary 2 Table S2: the sequences of mimics and siRNA used in cell transfection.

Supplementary 3 Table S3: putative target genes for let-7 g.
