# Supplementary material for: Grape Seed Procyanidin B2 Protects Porcine Ovarian Granulosa Cells against Oxidative Stress-Induced Apoptosis by Upregulating let-7a Expression
Source: Oxid Med Cell Longev. 2019 Nov 19;2019:1076512. doi: 10.1155/2019/1076512 (PMC6885843; doi:10.1155/2019/1076512)
Supplement: Supplementary 1 — Table S1: the forward and reverse primers used for qRT-PCR. [file 1076512.f1.docx]

Supplemental table S1 Primer sequences for real-time RT-PCR

| **Gene** | **Accession number** | **Primer sequences (5′- 3′)** | **Product**  **size**  **(bp)** | **Annealing temperature**  **(℃)** |
| --- | --- | --- | --- | --- |
| GAPDH | NM_001206359.1 | F: GGACTCATGACCACGGTCCAT | 220 | 58 |
|  |  | R: TCAGATCCACAACCGACACGT |  |  |
| Fas | NM_213839.1 | F: CGTGAGGGTCAATTCTGCTGT | 123 | 59 |
|  |  | R:CTTGTCTGTGTAATCCTCCCCTTC |  |  |
| Caspase-3 | NM_214131.1 | F: AATGGCATGTCGATCTGG | 353 | 58 |
|  |  | R: TCCCACTGTCCGTCTCAA |  |  |
