# Supplementary material for: Grape Seed Procyanidin B2 Protects Porcine Ovarian Granulosa Cells against Oxidative Stress-Induced Apoptosis by Upregulating let-7a Expression
Source: Oxid Med Cell Longev. 2019 Nov 19;2019:1076512. doi: 10.1155/2019/1076512 (PMC6885843; doi:10.1155/2019/1076512)
Supplement: Supplementary 2 — Table S2: the sequences of mimics and siRNA used in cell transfection. [file 1076512.f2.docx]

Supplemental table S2 The sequences of mimics and siRNA used in cell transfection

| **Mimics ID** | **Forward sequence (5’-3’)** | **Reverse sequence (5’-3’)** |
| --- | --- | --- |
| let-7a mimics | UGAGGUAGUAGGUUGUAUAGUU | CUAUACAACCUACUACCUCAUU |
| mimics NC | UUCUCCGAACGUGUCACGUTT | ACGUGACACGUUCGGAGAATT |
| let-7a inhibitor | AACUAUACAACCUACUACCUCA |  |
| inhibitor NC | CAGUACUUUUGUGUAGUACAA |  |
| siRNA-472 | CCAACCAGCAACACCAAAUTT | AUUUGGUGUUGCUGGUUGGTT |
| siRNA-822 | GCUGCUCCGUAAUUGGUAUTT | AUACCAAUUACGGAGCAGCTT |
| siRNA-942 | GGAUGUUACUAGUGAACAATT | UUGUUCACUAGUAACAUCCTT |
| NC-siRNA | UUCUCCGAACGUGUCACGUTT | ACGUGACACGUUCGGAGAATT |
